# Is an increase in skin temperature predictive of neuropathic foot ulceration in people with diabetes? A systematic review and meta-analysis

**DOI:** 10.1186/1757-1146-6-31

**Published:** 2013-08-07

**Authors:** Vanessa J Houghton, Virginia M Bower, David C Chant

**Affiliations:** 1 School of Surgery, University of Western Australia, Perth, Australia; 2 Statistical Consultant, Launceston, Australia; 3 M422 UWA Podiatric Medicine, 35 Stirling Highway, Crawley, WA 6009, Australia

**Keywords:** Diabetes, Foot complications, Ulceration, Temperature-monitoring, Prediction and prevention

## Abstract

**Introduction:**

The aim of this systematic review was to evaluate the strength of the existing research to answer the question: Is an increase in skin temperature predictive of neuropathic foot ulceration in people with diabetes?

**Methods:**

This study is a systematic review and meta-analysis of temperature-monitoring in the prediction and prevention of diabetic foot ulceration. Two investigators conducted a literature search for all relevant articles from 1960 until July 2011. During this process the following data bases were searched: MEDLINE, Science Direct, AMED, Australian Medical Index, APAIS-Health, ATSIhealth, EMBASE, Web of Science and OneSearch. Keywords used in this search included diabetes, foot complications, ulceration, temperature-monitoring, prediction and prevention.

**Results:**

Results of the meta-analysis support the theory that an increase in skin temperature is predictive of foot ulceration when compared with the same site on the contralateral limb. The theory that there is a mean norm foot temperature which can be used as a benchmark to monitor pathological change was unsupported by this meta-analysis.

**Conclusions:**

The conclusions derived from this review are based on the best available scientific evidence in this field. It is intended that the results of this study will improve clinical decision-making and encourage the appropriate measures used to predict and prevent ulceration in people with diabetes at high risk of foot complications. Based on quality studies in this area, the results of this review have indicated that the use of temperature-monitoring is an effective way to predict, and thus prevent, diabetic foot ulceration.

## Introduction and background

In the year 2000 it was estimated by the world health organisation that more than 171 million people in the world were suffering from diabetes mellitus. In this same study it was also predicted that, by the year 2030, this figure is likely to double [1].

Diabetic foot complications such as neuropathic ulceration are major contributors to morbidity and mortality and it is thought that 15% of people with diabetes will develop an ulcer at some point in their lives [2]. Foot complications in people with diabetes can be difficult to treat and conventional therapies often fail, leading to lower limb amputations. Thus, prevention of this condition is of paramount importance.

Interventions to prevent diabetic foot complications are numerous and varied. Foot examination by a clinician, custom footwear and orthoses, debridement of hyperkeratosis and offloading are just some of the preventative measures described in the literature. In addition to these methods, handheld skin temperature-monitoring has been found to be an effective monitoring instrument to reduce the incidence of foot complications, such as ulceration in people with diabetes [3-5].

The clinical signs of inflammation and soft tissue injury are often too subtle to be detected by the individual or even trained health care professionals. The five cardinal signs of inflammation include: pain, erythema, oedema, loss of function and heat. It is difficult to assess these subtle parameters, with the exception of skin temperature, which can be easily quantified by the layperson.

The concept of measuring skin temperature as a marker for inflammation and injury in the insensate foot was first addressed by Goller et al. in 1971, followed by Sandrow et al. in 1972 [6,7]. Goller reported a relationship between an increase in localised temperature and localised pressure whilst Sandrow used thermometry as a tool to diagnose neuropathic fractures [6,7]. Since that time there have been numerous studies evaluating this theory — many of which showed a positive relationship between inflammatory processes, tissue breakdown, and an increase in local skin temperature.

A literature review conducted in 2010 identified three types of temperature-measuring technologies that were available and used for the detection of foot complications [8]. Such technologies included: infrared thermometry; liquid crystal thermography; and temperature sensors integrated into weighing scales [8]. Only two of these methods—infrared thermometry and liquid crystal thermography—were included in this review. The method of using temperature sensors on weighing scales does not yet appear to have been assessed in the literature for its benefit in predicting or preventing ulceration in people with diabetes and hence there was no available data to include in this study [8].

Infrared thermography is a non-contact tool that detects the surface temperature at a particular point on an object [9]. These thermal images are useful when detecting temperature difference and quantifying sensitive changes in skin temperature that occur with pathological processes such as soft tissue inflammation and subsequent breakdown [9].

Liquid crystal thermography provides information about the distribution of temperature over the entire plantar surface of the foot through a coloured foot imprint on a plate comprised of layers of encapsulated thermochromic liquid crystals [8]. Warmth is transferred from the foot and accumulated in the plate, giving rise to a spectrum of colours representing temperature variations [8].

Both temperature monitoring techniques are useful, yet have flaws. Infrared temperature monitoring of the diabetic foot is quite time consuming. Liquid crystal thermography although faster, can be more difficult to interpret [8]. Infrared temperature monitoring does, however, stand out as the cheaper and more user friendly tool when the two are compared and is the technique which has been more widely adopted as the favoured method in recent times among diabetic foot practitioners [8].

In summary the aim of this systematic review was to evaluate the strength of the existing research to answer the question: Is an increase in skin temperature predictive of neuropathic foot ulceration in people with diabetes? Thermometry techniques evaluated in this study included liquid crystal thermography and infrared thermography.

## Methods

It is a well-established theory in medicine that prevention strategies are based on early detection and prediction of pathological change. Therefore, this review investigated both the prediction of diabetic foot ulceration through temperature-monitoring, and the relationship between temperature-monitoring and prevention of diabetic foot ulceration.

The question being asked in this systematic review and meta-analysis was placed under the category of ‘diagnostic test performance’— as described by the National Health and Medical Research Council (NHMRC) hand book on conducting systematic reviews [10]. These guidelines define this criterion as “How accurate a sign, symptom, or diagnostic test is in predicting the true diagnostic category of a patient”. In preparing this review, the NHMRC criteria for reviewing ‘diagnostic test performance’ were used as a guide.

### Selection of studies for inclusion

The types of studies selected for this review included both randomised and non-randomised trials, cohort studies, cross sectional studies and controlled trials.

Within the included studies there were three papers (Armstrong 1997a, Lavery 2004 and Armstrong 1997b) which involved participants with a Charcot arthropathy and neuropathic ulceration. The authors acknowledge that active Charcot arthropathy has an individual effect on local skin temperature which resides during the quiescent phase of the condition. Skin temperature monitoring is used to monitor active Charcot arthropathy in the same manner as it is used to monitor neuropathic ulceration, however, active Charcot arthropathy was a condition excluded from this review as the primary research question focused on neuropathic ulceration.

For this reason only the data which related to cases with neuropathic ulceration occurring in the quiescent Charcot arthropathy phase were included for analysis from the Armstrong 1997a study. For the Lavery 2004 study, data for the Charcot arthropathy cases were excluded from the analysis. A new odds ratio was calculated using only the data of participants with neuropathic ulceration. Within the Armstrong 1997b study the Charcot arthropathy cases were reported and analysed as a separate cohort to the neuropathic ulceration group. For the purposes of this review only the neuropathic ulceration participant data in this study was used for analysis.

### Publication bias

Two investigators conducted a literature search for all relevant articles from 1960 until July 2011. The Cochrane library was searched initially, to ensure no systematic reviews had previously been conducted on this topic. Then a search was conducted on relevant articles using keywords and synonyms of: diabetes, foot complications, ulceration, temperature-monitoring, infrared thermometry, self-monitoring, and prevention. During this process the following data bases were searched:

•MEDLINE

•Science Direct

•AMED

•Australian Medical Index

•APAIS-Health

•ATSIhealth

•EMBASE

•Web of Science

•OneSearch

Citations within obtained articles were hand-searched and scrutinised to identify additional studies. A list of clinical trial data bases was obtained from the Cochrane handbook. The relevant data bases on this list were searched for completed published, unpublished and ongoing unpublished studies. Conference proceedings were examined using the Index to Scientific and Technical Conference Proceedings. Attempts to access the grey literature were made using the Health Management Information Consortium database and System for Information on Grey Literature. Attempts to access articles written in languages other than English were made using the Virtual Health Library and articles with abstracts scrutinised. All studies deemed eligible for inclusion into this review were cross checked to exclude the risk of duplicate publication inclusion.

In order to reduce the possibility of a positive study publication bias, the reviewers’ best efforts were made to recover all unpublished and grey literature on the area of interest. For time and resource reasons, expert authors in the area of diabetic foot complications and temperature-monitoring were not contacted and questioned about further unpublished or grey literature that may exist.

The selection of papers to be integrated in a meta-analysis has, of course, a bearing on the conclusions reached, and perhaps the greatest problem in meta-analysis is publication bias. An informal method of assessing publication bias is the so-called funnel plot. The funnel plot assumes that results of smaller studies will be more widely spread about the mean effect because of larger random error. A plot of a measure of "size", i.e. the precision (often measured by inverse standard error) of the papers vs treatment effect should therefore be shaped like a funnel if there is no publication bias. Funnel plots were performed on all meta-analyses in this review.

### Selection bias

The NHMRC guidelines [10] state that the ideal design of a prognostic study is a cohort study. The ‘inception’ cohort of people with a condition should be followed for a sufficiently long period of time for major outcomes to have occurred. For these reasons, studies were included in this review according to their quality as cohort studies. Issues of randomisation and blinding of investigators who enrolled study participants were not considered when selecting appropriate studies. Including such criteria in the selection of cases would have reduced the number of studies in the prediction meta-analysis to only one.

The checklist developed by the Cochrane Methods Working Group on ‘Screening and Diagnostic Tests’ (sourced from the NHMRC [10]) was used to assess the quality and applicability of studies. All studies met all of the criteria for inclusion in this review according to this checklist.

## Results

See Table 1 for characteristics of included studies.

**Table 1:** Characteristics of included studies

**Reference**	**Title**	**Objective**	**Study design**	**Population**	**Temperature-monitoring**	**Sites measured**	**Temperature control**	**Outcome**
Armstrong 1996	Monitoring Neuropathic Ulcer Healing with Infrared Dermal Thermometry.	To prospectively evaluate skin temperatures at the site of neuropathic ulceration before, during, and after wound healing using the contralateral extremity as a physiologic control and to evaluate variables that may influence skin temperature gradients.	Longitudinal.	Location: USA	Foot temperatures were taken at weekly cast changes and for the first four months after return to shoes.	The anatomical site of ulceration.	Contralateral limb was used as control.	Skin temperature gradient was a mean of 6.9°F higher on the ulcerated site than at the same anatomical site on the contralateral limb when the initial total contact cast was applied (91.1 vs 84.2°F). At the end of total contact casting, Armstrong 1996 found no significant difference in skin temperature between the previously ulcerated site and the same site on the contralateral limb (83.4 vs 85.3°F).
Group 1				Number: 25				
				Age: mean 52.4, SD 11.6	Temperatures were taken using a hand-held, infrared skin temperature probe (Exergen DT 1001).			
				Groups: All patients had grade I ulcerations. All patients had loss of protective sensation.				
					Temperatures were also measured at 2, 4 and then 8 week intervals after transfer to shoe gear for four months following return to shoe gear.			
Armstrong 1997a	Monitoring Healing of Acute Charcot’s Arthropathy with infrared Dermal thermometry.	Describe the use of skin temperature assessment in diabetics with acute Charcot's arthropathy to monitor resolution of inflammation longitudinally throughout the course of treatment and to predict development of neuropathic ulcers.	Longitudinal.	Location: USA	Total contact casts changed weekly in patients with ulcerations. No ulcers (changed after 3 weeks at most).	The anatomical site of ulceration.	Contralateral limb was used as control.	There was no significant difference in skin temperature gradients on initial presentation between ulcerated and non- ulcerated subjects (1.5+/−1.1 vs 2.2+/−1.4°F). However, all patients in the study had acute Charcot’s arthropathy at this time.
				Number: 39				
Group 1				Age: mean 59, SD 9.5				
				Groups: All patients had acute Charcot’s arthropathy, diabetes, loss of protective sensation and palpable pedal pulses.	Measured temp on seven sites on sole of the foot and on the anterior ankle.			During the follow-up period of this study, three participants with inactive Charcot’s arthropathy had ulcerated under the forefoot. At their visits prior to re-ulceration, temperature gradients between the ulcerated and non-ulcerated foot were significantly higher than those of other patients seen at that clinic (4.5+/−0.9 vs 0.9+/−0.9°F).
				17 patients initially presented with concomitant grade 1A ulceration also.				
Armstrong 1997b	Infrared Dermal Thermometry for the High-Risk Diabetic Foot.	Compare skin temperatures in patients with asymptomatic peripheral sensory neuropathy, patients with neuropathic ulcers, and patients with Charcot’s arthropathy using the contra-lateral limb as control.	Cohort study.	Location: USA	Temperatures were taken using a hand-held, infrared skin temperature probe (Exergen DT 1001).	Great toe, the first metatarsocuneiform joint, the cuboid and the first, third and fifth metatarsal heads.	Contralateral limb.	There were differences in skin temperature between the affected foot and the contralateral foot among the patients with neuropathic ulcers (3.1°C) patients with neuropathic ulcers experienced re-ulceration a mean of 12.2 months after initial healing, with a corresponding increase in skin temperature 3.2°C vs. 28°C at the clinical visit preceding injury.
				Number: 143				
Group 1				Mean age: 63.9	Temps were taken by the study physician when casts were checked (no longer than 3 weeks).			
				Groups – Asymptomatic sensory neuropathy (n=78), Ulcers (n=44), Charcot (n=21).				
Armstrong 2007	Skin Temperature-monitoring Reduces the Risk for Diabetic Foot Ulceration in High-risk Patients.	To evaluate the effectiveness of home temperature-monitoring to reduce the incidence of foot ulcers in high-risk patients with diabetes.	Randomised controlled trial.	Location: USA	Both groups involved therapeutic footwear, diabetic foot education, regular foot care and a structured daily foot inspection.	Six foot sites (not reported in the article).	Contralateral limb.	Treatment and control groups demonstrated no significant differences in descriptive characteristics (p>0.05). Total of 8.4% ulcerated (n=19). 12.2% (n=14) ulcerated in the standard therapy group. 4.7% (n=5) ulcerated in the dermal thermometry group. Ulcerating patients had a temp difference that was 4.8 times greater in the region of the ulceration.
Group 1 and Prevention				Number: 225				
				Mean age: 69				
				Groups: Standard therapy (n=115) Dermal thermometry (n=94).	The dermal thermometry also involved skin temperature-monitoring using the TempTouch device.			
					Temperature difference >4°F between left and right corresponding sites triggered patients to contact the study nurse and reduce activity until temperature normalised.			
Armstrong 2003	Skin Temperatures as a One-time screening Tool Do Not Predict Future Diabetic Foot Complications.	Are baseline mean skin temperature measurements useful in predicting the most common foot-related complications of diabetes mellitus?	Prospective longitudinal study.	Location: USA	Temperatures were taken using a hand-held, infrared skin temperature probe (Exergen DT 1001).	Great toe, the first metatarsocuneiform joint, the cuboid and the first, third and fifth metatarsal heads. All 12 measurements were averaged.	Participants in the study who did not ulcerate.	There was no difference in the mean foot temperature of participants who had ulcers compared to participants who did not have ulcers during the study.
Group 2				Number: 1588				
				Mean age: 69				
				Groups: number of variable groups. This meta-analysis only used data of patients who developed ulcerations and those who did not.				
Benbow 1994	The Prediction of Diabetic Neuropathic Plantar Foot Ulceration by Liquid-Crystal Contact Thermography.	To assess whether the development of plantar foot ulceration could be predicted from the mean foot temperature (MFT) as assessed by liquid crystal thermometry in patients with peripheral neuropathy.	Cohort study.	Location: UK	Temperatures were taken using liquid crystal thermometry.	First to fifth metatarsal heads, navicular, cuboid and plantar heel. All 16 measurements were averaged.	Non-diabetic paticipants.	Without PVD participants: At follow-up seven feet had developed ulcerations. The initial MFT in the seven feet that developed plantar ulcers (30.5+/− 2.6C) was significantly higher than in the 38 feet that did not develop ulcers (27.8 +/− 2.3C).
Group 2				Number: 50				
				Mean age: 56.3				
				Groups: Diabetic neuropathic peripheral vascular disease (PVD), diabetic neuropathic without PVD and non diabetic non PVD.				
								With PVD participants: No results available.
Stess 1986	Use of Liquid Crystal Thermography in the Evaluation of the Diabetic Foot.	Identify thermal emission patterns associated with diabetic foot ulcers.	Case control. 3 groups.	Location: USA	Liquid crystal thermography.	Entire plantar surface.	Non-diabetic participants.	In patients with unilateral ulcerations, no significant temperature difference was detected between the affected and unaffected feet under the met heads, hallux of heels.
Group 2				Number: 65				
				Mean age – not indicated				
				Groups: Group 1 – no history of diabetes (16), Group 2 – no history of, or active foot ulceration (21) Group 3 – active or history of plantar ulceration (28) 13 presented with active ulceration.				
Lavery 2007	Preventing Diabetic Foot Ulcer Recurrence in High-Risk Patients.	Evaluate the effectiveness of a temperature-monitoring instrument to reduce the incidence of foot ulcers in individuals with diabetes who have a high risk for lower extremity complications.	Randomised controlled trial.	Location: USA	Temperatures were taken using a hand-held, infrared skin temperature probe (Exergen DT 1001).	The great toe, the first, third and fifth metatarsal heads, the mid-foot and the heel.	Contralateral limb.	Fewer ulcers developed in the enhanced therapy group than the standard therapy group and the structured foot examination group (enhanced therapy 8.5 vs. standard therapy 29.3%, p = 0.0046 and enhanced therapy vs. structured foot examination 30.4%, p = 0.0029). Patients in the standard therapy and structured foot examination were 4.37 and 4.71 times more likely to develop ulcers than patients in the enhanced therapy group.
Prevention				Number: 173				
				Age: mean 64.9	Each group received therapeutic footwear, diabetic foot education and regular foot care. Subjects in the structured foot examination group performed structured foot examinations daily. Patients were to contact the nurse if they noted any abnormalities. Subjects in the enhanced therapy group measured foot temperatures on six sites, daily. If a temperature difference of 4°F was noted between limbs, patients were to inform the study nurse.			
				M:F: 93:80				
				Groups: Three groups – standard therapy, enhanced therapy and structured foot examination.				
Lavery 2004	Home Monitoring of Foot Skin Temperatures to Prevent Ulceration.	To evaluate the effectiveness of at-home infrared temperature-monitoring as a preventative tool in individuals at high risk for diabetes-related lower-extremity ulceration and amputation.	Randomised controlled trial.	Location: USA	Temperatures were taken using a hand-held, infrared skin temperature probe (Exergen DT 1001).	The great toe, the first, third and fifth metatarsal heads, the central mid-foot and the heel.	Contralateral limb.	The enhanced therapy group had significantly fewer foot complications (enhanced therapy group 2% vs. standard therapy group 20%, p = 0.01, odds ratio 10.3, 95% CI 1.2-85.3). There were seven ulcers and two Charcot fractures among standard therapy patients and one ulcer in the enhanced therapy group.
Prevention				Number: 85				
				Age: mean 54.9	Standard therapy involved therapeutic footwear, diabetes foot education and regular foot evaluation by a podiatrist. Enhanced therapy included the addition of a handheld infrared skin thermometer to measure temperatures n the sole of the foot in the morning and evening. When skin temperature differences were more than 4°F.			
				M:F: 43: 42				
				Groups: Standard therapy and enhanced therapy group.				

**Figure 1:**
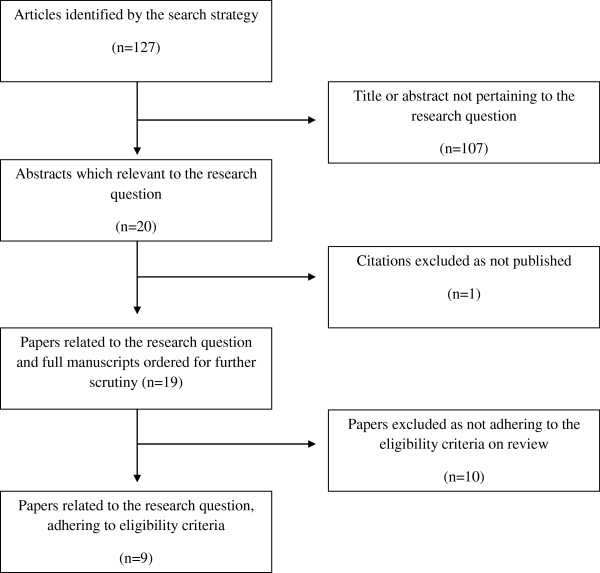
Quorum Flowchart of the Reviewing Process.

Nineteen studies, as illustrated in Figure 1 were identified in the literature search and deemed eligible for further scrutiny. Of these nineteen studies, nine met the inclusion criteria for this review and were eligible for qualitative and statistical analysis. This review incorporates two subtopics: a study which assesses temperature-monitoring as a predictive tool for diabetic ulceration; and a study that assesses temperature-monitoring as a tool aiding in the prevention of diabetic ulceration. Seven studies were included in the prediction section of the review and three studies were included in the prevention section of the review. One study was deemed eligible for inclusion into both of the aforementioned sections of the review.

### Prediction

There were eight mean temperature differences from seven studies included in the meta-analysis which focused on the use of temperature-monitoring as a predictive tool for diabetic foot ulceration. Within these seven studies, there were two study design groups. Studies classified as group “one” compared the temperature between the same anatomical sites on contralateral feet. Studies classified as group “two” included those that assessed mean skin temperature of both feet.

### Group “One” results

Articles included in group “one” of the ulcer prediction meta-analysis included: Armstrong 1996, Armstrong 1997a and 1997b and Armstrong 2007 [5,11-13]. Armstrong 1996 found the skin temperature gradient to be a mean of 6.9°F higher on the ulcerated site than at the same anatomical site on the contralateral limb when initial total contact cast was applied (91.1°F vs 84.2°F, t = 8.9, p<0.0001, 95% CI 5.3, 8.5). At the end of total contact casting, Armstrong 1996 found no significant difference in skin temperature between the previously ulcerated site and the same site on the contralateral limb (83.4°F vs 85.3°F, t = −1.35, NS, 95% CI −7.5, 3.9). This longitudinal study monitored participants with neuropathic ulcerations throughout the healing process to determine temperature gradient trends between the ulcerated and non-ulcerated limb.

Armstrong 1997a found temperature gradients to be significantly higher in the neuropathic ulceration participants than those of other patients seen at that clinic (4.5+/−0.9°F vs 0.9+/−0.9°F). This was the result used in this meta-analysis. This longitudinal study followed participants who were at high risk of developing ulcers and monitored pre-ulcerative states in participants who exhibited an increase in temperature prior to soft tissue breakdown.

Similarly, Armstrong 1997b found that temperatures were higher on the ulcerated foot than the same site on the contralateral foot on initial presentation with an ulcer (5.6°F p< 0.0001). Temperatures were the same between contralateral sites after healing. In this study, 11.4% of patients experienced re-ulceration at the site of previous ulceration. Skin temperatures taken at the visit prior to re-ulceration were higher on the pre-ulcerative limb than at the same site on the contralateral limb (89.6 +/− 1.2°F vs 82.5+/− 2.9°F, p = 0.003). In common with Armstrong 1996, this longitudinal study monitored skin temperature gradients during ulcer healing as well as monitoring for a relationship between skin temperature change and re-ulceration.

Furthermore, Armstrong 2007 noted the mean temperature difference at the site of ulceration to be 4.8 times greater, one week before ulceration, than in a group of control patients (3.50+/− 1.0°F vs 0.74 +/− 0.05°F, p = 0.001). This study was a very well designed randomised controlled trial which focused on the use of temperature monitoring in the prevention of ulceration. This study identified a positive relationship between increased skin temperature and the pre-ulceration phase.

In conclusion, all four studies in group “one” of the prediction meta-analysis discovered a relationship between an increase in temperature gradient between the site of ulceration and the same site on the contralateral foot. Although all four studies took a different methodological approach — they were all able to be analysed collectively to reach the same conclusion.

### Group “Two” results

Studies classified as group “two” included Stess 1988, Benbow 1994 and Armstrong 2003 [14-16]. Stess 1988 compared mean temperatures between the ulcerated foot and the contralateral, non-ulcerated foot. No statistically significant mean temperature difference was found between feet, under the metatarsal heads, the great toe or the heel on the ulcerated vs the non-ulcerated limb. A case-controlled design was adopted for this study, a design suitable for answering the question of whether mean foot temperatures were indicative of neuropathic ulceration.

Benbow 1994 presented two results relevant to this review: comparisons between participants with peripheral vascular disease vs control participants; and participants without peripheral vascular disease vs control participants. The results of these analyses found that participants with high plantar foot temperatures were at increased risk of neuropathic foot ulceration. A cohort study design was used by Benbow in this paper to compare groups or patients who ulcerated with those who did not.

Armstrong 2003 concluded that measurement of non-focal mean skin temperature is not an effective means of screening people for future diabetic ulceration. There was not a statistically significant difference between the mean baseline temperatures of participants that did and did not ulcerate (83.1 +/− 4.9°F vs 82.5 +/− 4.6°F; p = 0.06). These conclusions were ascertained using a longitudinal design in which participants were followed for a two year period during which ulcer occurrence was monitored and recorded.

The figures from groups “one” and “two” were separately and collectively statistically analysed.

### The relationship between group “One” and group “Two” results

For the eight measures of temperature difference used in this analysis, the test statistic for heterogeneity takes the value 301.7 which, with a chi-squared distribution of seven degrees of freedom, is highly statistically significant. Applying the random effects model to the data gives a pooled estimate of the population temperature difference of 3.36°F with a 95% confidence interval of (1.86, 4.86). See Table 2 for detailed results and for forest plot representation of data.

**Table 2:** Summarised mean temperature differences between studies classified as group “one” and group “two” (°F)

**Group**	**Sample size**	**Mean temperature**	**95% confidence**	**95% confidence**
		**difference**	**limit (lower)**	**limit (upper)**
1	4	4.88	2.80	6.96
2	4	1.43	−0.44	3.30
difference		3.45	0.77	6.14

**Figure 2:**
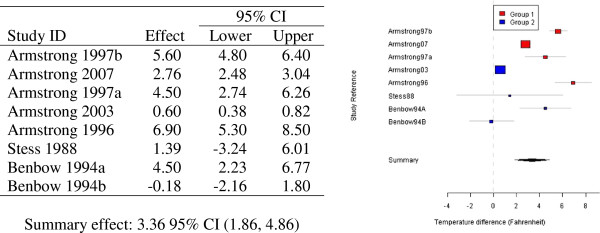
Random effects modelling of eight mean temperature differences in the “Effect” column (°F) and associated forest plot of observed mean temperature differences (°F, 95% CI) for groups “one” and “two”.

A mean difference of 3.45 was found when comparing the two covariate groups using weighted linear regression. The lower 95% confidence limit of 0.77 is above zero, corresponding to a statistical hypothesis test of no treatment difference having a p-value of less than 0.05, a value which is usually taken as moderate evidence against the associated null hypothesis. These results are summarised in Figure 2.

### Publication bias

**Figure 3:**
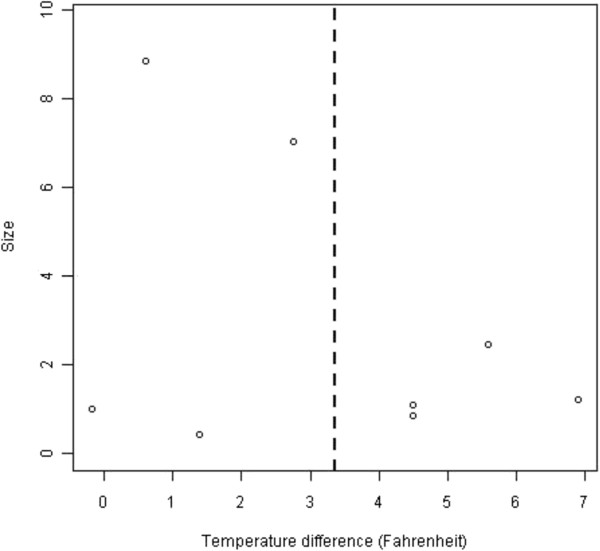
A funnel plot of observed mean temperature differences (°F, 95% CI).

For the eight mean foot temperatures in the seven articles analysed, a funnel plot was constructed. The plot is given in Figure 3, and is consistent with, but far from a conclusive demonstration of, a lack of publication bias.

### Prevention

All three studies included in the prevention analysis of this review were written by authors, Armstrong and Lavery. All three studies were homogenous in nature, making the conduction of statistical tests a simple task. All three studies were well-designed randomised controlled trials with physician blinding. They all compared the incidence of ulceration in subjects who monitored their foot temperatures daily, with those who did not. In all three studies there was a clear association between temperature-monitoring and reduced ulceration rate.

Lavery 2007 established three subject groups, two of which did not monitor foot temperature, and one which did monitor foot temperatures on the same anatomical sites on each foot [4]. The incidence of foot ulceration during the 15 month evaluation period was very similar between the two groups that did not use temperature-monitoring (29.3% and 30.4%) compared to 8.5% of subjects in the temperature-monitoring group who developed ulcerations [4].

Lavery 2004 found, that of the 84 subjects included in their study, 20% of the 44 subjects in the standard therapy group presented with diabetic foot complications. This was compared to the temperature-monitoring group, in which only one subject (2%) ulcerated.

In the third study, Armstrong 2007 found subjects were one third as likely to ulcerate in the skin temperature-monitoring group compared with the control group (12.2% vs 4.7%, OR 3.0, 95% CI, 1.0, 8.5, p = 0.001). This study performed a proportional hazards regression analysis which suggested that temperature-monitoring is associated with a significantly longer time to ulceration (p = 0.04).

Two meta-analyses for the three papers discussed above were undertaken. A total of four odd-ratios on ulceration for control group vs temperature-monitoring group were conducted. Lavery 2007 presents two odds-ratios, one for each type of standard therapy group vs temperature-monitoring group and structured foot examination group vs temperature-monitoring group, (OR 4.48, 95% CI, p = 0.008) and (OR 4.71, 95% CI, p = 0.006) respectively.

**Figure 4:**
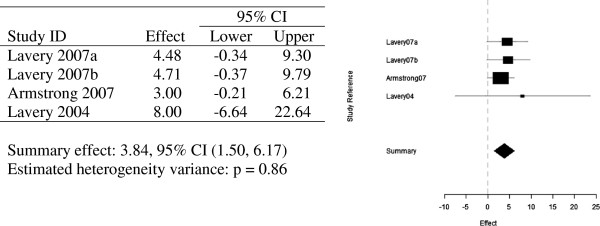
Random effects meta-analysis - four odds ratios and forest plot of observed odds ratios and 95% confidence intervals for the prevention studies.

Lavery 2004 presented a much greater odds-ratio than the other papers (OR 8.00, 95% CI). The odds on ulceration among the control group are eight times the corresponding odds on the temperature-monitoring group, but with a much wider confidence interval, than the other three odds-ratios. This can be visualised in Figure 4.

**Figure 5:**
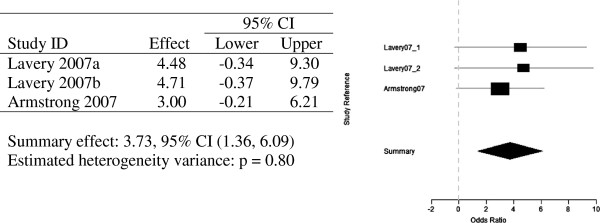
Random effects meta-analysis of three odds ratios (Lavery 2004 omitted) and forest plot of observed odds ratios and 95% confidence intervals for the prevention studies (Lavery 2004 omitted).

Meta-analysis of these three articles combined concluded a summary effect of 3.80 with a 95% confidence interval. The wider confidence interval of Lavery 2004 contributes much less weight to the summary effect of 3.80 than the other three odds-ratios analysed in this meta-analysis. This is made even clearer in Figure 5, in which Lavery 2004 is omitted, and the summary effect only drops to 3.73. The odds ratio being greater than one in this meta-analysis, thus indicates, that subjects who participated in the daily monitoring of their foot temperatures were at a lesser risk of developing ulceration.

### Publication bias

**Figure 6:**
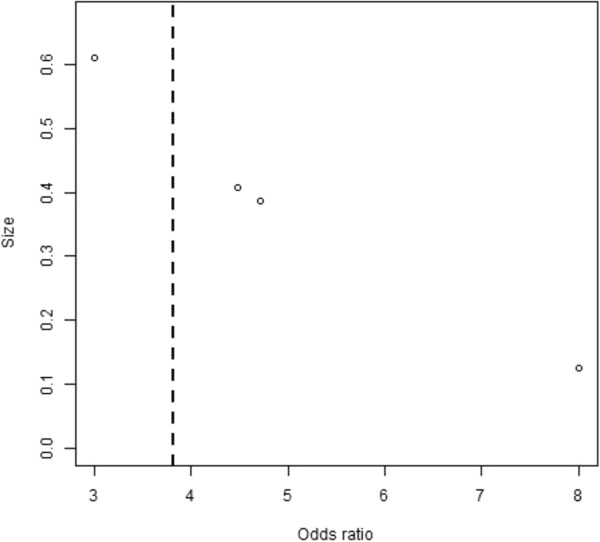
A funnel plot of odds ratios from the three prevention articles.

For the four odds ratios from the three prevention articles analysed in this review, a funnel plot was constructed (Figure 6). This plot indicates a lack of publication bias.

## Discussion

Studies included in this review were of appropriate design to address their aims and objectives. This was established using the National Health and Medical Research Council (NHMRC) hand book on conducting systematic reviews. Study designs within this systematic review included three well designed randomised controlled trials, three longitudinal studies, two cohorts and a case-controlled study.

The findings of this review show a clear correlation between an increase in foot temperature and subsequent foot ulceration. This review also supports the proposition that daily skin temperature-monitoring can contribute to preventing the development of diabetic foot ulceration.

The meta-analysis confirms that there was a significant temperature difference between ulcerated sites and the corresponding site on the contralateral foot in the group “one” data. However, the method of prediction of ulceration used in group “two” was not supported by this review. All four group “one” studies employed infrared temperature measurement devices and two of the three group “two” studies used liquid crystal thermography. Liquid crystal thermography has been described as a less accurate tool than infrared thermography. This may have influenced the accuracy of the results of the liquid crystal studies and potentially lessened the value of the comparison between the two groups.

This review did not address the use of temperature-monitoring as a predictive and preventative tool for Charcot’s arthropathy. Charcot’s arthropathy is a devastating complication of diabetes that often leads to severe deformity and permanent disability [17,18]. Studies investigating the relationship between an increase in temperature and the development of Charcot’s arthropathy were excluded from this review. These articles were excluded as the volume of published data was too low to undertake a quality systematic review and meta-analysis.

A further limitation of this study is that the authors were unable to contact authors of included studies to complete the literature search. For future research it would be valuable to ensure adequate time and resources are available to ensure that this stage of the research process was thorough and complete.

The results of this review indicate that the more contemporary technique of comparing temperature change between the same anatomical sites on each foot is superior. From the results of this study it is also reasonable to suggest that there is insufficient evidence to support the theory that there is an absolute-norm skin temperature which is predictive of diabetic foot ulceration.

## Conclusions and recommendations for future research

This review has made its conclusions based on the best available scientific evidence in this field. It is intended that the results of this study will improve clinical decision-making and encourage appropriate measures to be used to predict and prevent ulceration in people with diabetes at high risk of foot complications. Based on quality studies in this area, the results of this review have indicated that the use of temperature-monitoring is an effective way to predict, and thus prevent, diabetic foot ulceration.

Skin temperature change and its relationship with Charcot arthropathy was not analysed in this study for reasons previously described in the discussion section of this paper. The authors’ of this paper do, however, acknowledge the clinical relevance of this association and recommend that future research into skin temperature monitoring should focus on investigating the relationship between these two variables. Podiatric clinicians are routinely using skin temperature-monitoring as a clinical tool to monitor the acute phase of Charcot’s arthropathy and yet there may be a question about the strength of the association between a temperature change and destructive activity associated with Charcot’s.

The cost of temperature-monitoring could also prove to be a suitable topic for future research as there is currently no published literature available on this topic. A review of the cost of diabetes complications in Australia, Canada, France, Germany, Italy and Spain identified that treatment costs for both infected and uninfected ulcers were very high and reasonably consistent across the three identified countries [19]. Treatment of an infected ulcer was highest in Australia 3105AUD and France 2551AUD, followed by Germany 2275 AUD and Canada 1941 AUD [19]. The findings of this systematic review indicate that temperature-monitoring is a cost-effective tool for the prevention of foot ulcers if used appropriately and in the right patient-risk classification group.

Two temperature monitoring techniques, infrared dermal thermometry and liquid crystal thermometry were investigated in this systematic review. Both techniques have been found to be effective in previous studies, however, through this review it has been determined that infrared is the more widely used, user friendly and cost-effective of the two tools.

One of the primary endeavours of health care research is to provide robust evidence to support clinical practice. The purpose of this review was to determine whether or not a commonly used technique in the prevention of foot ulceration was supported with sufficient evidence to endorse the practice. The authors of this review are confident that both the qualitative and the quantitative results of this study provide sufficient evidence to support the continued use of skin temperature monitoring between comparative anatomical sites in the prevention of foot ulceration.

In summary we conclude that infrared temperature monitoring is an important tool in the prevention of foot ulceration in people with diabetes. Furthermore, the evidence reviewed for this study supports the role of self-care in prevention of foot ulceration. Three articles included in this review demonstrated the effectiveness of home monitoring in preventing neuropathic foot ulceration. In Australia skin temperature monitoring is primarily used in hospital high risk foot services. There is clearly a preventative role for self-monitoring of foot skin temperature among individuals with high risk feet as evidenced in this review. It is hoped that the conclusions of this review will encourage community health practitioners and public health services to embrace the preventative value of this method with confidence.

Diabetes related foot ulceration presents a significant personal, social and economic burden worldwide. The identification of cheap, simple and evidenced based tools to predict and prevent foot ulceration — such as temperature monitoring — has the potential to significantly reduce this burden. The authors of the studies included for analysis in this review are acknowledged and applauded for the important contribution they have made to the field of diabetic foot medicine.

## Competing interests

The authors declare that they have no competing interests.

## Authors’ contributions

VH and VB conducted the initial literature search. VH and VB conducted the systematic review. VH and VB wrote up and edited the manuscript, a larger portion was written and edited by VH. DC conducted the meta-analysis. All authors read and approved the final manuscript.
